# Generation of a Conditional Allele of the Transcription Factor Atonal Homolog 8 (Atoh8)

**DOI:** 10.1371/journal.pone.0146273

**Published:** 2016-01-11

**Authors:** Miriam Ejarque, Joan Mir-Coll, Ramon Gomis, Michael S. German, Francis C. Lynn, Rosa Gasa

**Affiliations:** 1 Diabetes and Obesity Research Laboratory, Institut d’Investigacions Biomèdiques August Pi i Sunyer (IDIBAPS), Barcelona, Spain; 2 Centro de Investigación Biomédica en Red de Diabetes y Enfermedades Metabólicas Asociadas, Barcelona, Spain; 3 University of Barcelona, Barcelona, Spain; 4 Department of Medicine, Diabetes Center, University of California, San Francisco, United States of America; 5 Diabetes Research Program, Child and Family Research Institute, Vancouver, Canada; 6 Department of Surgery, University of British Columbia, Vancouver, Canada; Vrije Universiteit Brussel, BELGIUM

## Abstract

Atonal Homolog 8 (Atoh8) is a basic helix-loop-helix (bHLH) transcription factor that is highly conserved across species and expressed in multiple tissues during embryogenesis. In the developing pancreas, Atoh8 is expressed in endocrine progenitors but declines in hormone-positive cells, suggesting a role during early stages of the endocrine differentiation program. We previously generated a whole-body *Atoh8* knockout but early lethality of null embryos precluded assessment of *Atoh8* functions during organ development. Here we report the generation of a conditional *Atoh8* knockout mouse strain by insertion of two loxP sites flanking exon 1 of the *Atoh8* gene. Pancreas-specific *Atoh8* knockout (Atoh8 Δ^panc^) mice were obtained by mating this strain with a Pdx1-Cre transgenic line. Atoh8 Δ^panc^ mice were born at the expected mendelian ratio and showed normal appearance and fertility. Pancreas weight and gross pancreatic morphology were normal. All pancreatic cell lineages were present, although endocrine δ (somatostatin) cells were modestly augmented in Atoh8 Δ^panc^ as compared to control neonates. This increase did not affect whole-body glucose tolerance in adult knockout animals. Gene expression analysis in embryonic pancreases at the time of the major endocrine differentiation wave revealed modest alterations in several early endocrine differentiation markers. Together, these data argue that Atoh8 modulates activation of the endocrine program but it is not essential for pancreas formation or endocrine differentiation in the mouse. Given the ubiquitous expression pattern of Atoh8, the availability of a mouse strain carrying a conditional allele for this gene warrants further studies using temporally regulated Cre transgenic lines to elucidate time or cell-autonomous functions of Atoh8 during development and in the adult.

## Introduction

Atonal Homolog 8 (Atoh8) is a basic helix-loop-helix (bHLH) transcription factor highly conserved across species and expressed in multiple organs during embryonic development [1–5]. Atoh8 is thought to participate in differentiation programs even though its precise molecular functions remain largely unknown. The *Atoh8* gene is upregulated by ectopic expression of several lineage-determining bHLH factors in cultured cells, indicating a potential general role of Atoh8 in differentiation programs driven by bHLH proteins [3, 6–8]. Thus far Atoh8 has been involved in the specification and differentiation of neuronal lineages, favoring neurogenesis over gliogenesis [4]. Furthermore, Atoh8 is expressed in several neuronal subtypes in the adult brain, indicating a function in the maintenance of these cellular subtypes besides of its roles in the determination of neuronal fate. In mice, Atoh8 has been involved in kidney and liver development [2, 9]. It has also been implicated in the development of retina and skeletal muscle in chicken and zebrafish [1, 5, 10]. In humans, ATOH8 has been shown to contribute to shear stress stimulated endothelial differentiation during embryonic endothelial development [11], to participate in muscle fiber regeneration [12] and to repress stem cell genes in hepatocellular carcinoma cells [13].

Given the ubiquitous expression pattern and multiple roles ascribed to Atoh8, a conditional gene knockout mouse model would be highly valuable to decipher its function in specific tissues and cell lineages at precise developmental stages. Here we describe the generation of a conditional *Atoh8* knockout mouse line by flanking the *Atoh8* exon1 with loxP sites (flox). To verify that this allele can be deleted *in vivo* by the Cre recombinase in a tissue-specific manner, *Atoh8* floxed mice were mated with a Pdx1-Cre line [14] to generate pancreas-specific *Atoh8* knockouts. We had previously identified Atoh8 as a component of the embryonic pancreas transcriptional network, but due to early lethality of knockout embryos, its function during pancreatic development *in vivo* could not been addressed [3].

## Methods

### Construction of the targeting vector

The region (~-8->~+3.5kb) surrounding the Atoh8 translational start site was gap repaired from a BAC containing the entire *Atoh8* gene (RP22-157F13; 129S6/SvEvTac) into a PGK-TK containing Bluescript vector using homology arms bounded by the following primers (5’F-GCCACTCCTCCTGCATTTTCTGTTAC; 5’R-CCATCTCCTCATGCCCTGTCAG; 3’F-TCAGGTTGCATCATGACGTTATCCTC; 3’R- GCCAGAGTTCGATCCCCA AG). The PGK-TK was placed downstream of the shorter 3’ homology arm. 5’ and 3’ LoxP sites were then introduced using recombineering and the pL452 and pL451 vectors respectively and the first selection cassette was then removed using Cre-expressing EL350 E Coli as previously described [15]. The 5’ LoxP site was inserted in the *Atoh8* upstream region as indicated: ATCAATTGTTATCATTCCCAGGAGGA-LoxP-AGGTGTGGTTGTGACCCCTATCCT. The 3’ LoxP site was inserted into intron 1 as indicated CAGATGACAGAGGGCAGGGAGTTG-LoxP-CCTGTATATCTGCTTTGCTTGTGGTG. The final construct contained an FRT flanked PGK-promoter driven NeoR gene, that is part of pL451, for selection and subsequent excision using FLPe.

### Generation of the *Atoh8* floxed allele

The *NotI* linearized targeting vector was electroporated into E14 mouse embryonic stem cells by the UCSF transgenic core. After standard selection with G418 and ganciclovir (Ganc), correctly targeted ES cell clones were screened by PCR and then Southern blot analysis using external probes. Chimera mice were generated from three correctly targeted ES clones by microinjection into C57BL/6J blastocysts. Germline transmission was achieved by breeding to C57BL/6J females and targeting confirmed using Southern blotting on EcoNI and XmnI digested genomic DNA. The PGK-neo selection cassette was then removed by crossing with Actin-Flpe mice (Jax #005703) and confirmed by PCR. Subsequent genotyping of the floxed Atoh8 mice was carried out by PCR to detect LoxP inclusion (primers in S1 Table). Pancreas-specific Atoh8 knockout mice (Atoh8 Δ^panc^) were generated by crossing floxed *Atoh8* with the Pdx1-Cre strain. Genomic PCRs were done on DNAs isolated from tail tissues.

### Mice

Pdx1-Cre mice were described elsewhere [14]. Mice carrying the Cre transgene and the conditional *Atoh8* allele, generated at the University of California at San Francisco, were sent to IDIBAPS for phenotypic characterization. All mice were bred and maintained at the barrier animal facility of the University of Barcelona. Embryonic tissues were collected at indicated times, considering the morning of the appearance of a vaginal plug as embryonic day (E)0.5. Principles of laboratory animal care were followed (European and local government guidelines) and animal procedures were approved by the Animal Research Committee of the University of Barcelona. Animals were euthanized by cervical dislocation.

### RNA extraction and qRT-PCR

Total RNA was isolated from embryonic pancreases using the RNeasy MicroKit (Qiagen, Hilden, Germany) and quantified using a Nanodrop 1000 (Thermo Scientific, Wilmington, MA). RNA was reverse-transcribed using the Superscript III Reverse Transcriptase (Invitrogen) following the manufacturer’s instructions. Real time PCR was carried out in ABI7900 cycler using a Sybr green master mix (Express Greener, Invitrogen). mRNA expression levels were normalized to the expression of the Tata binding protein (*tbp*) for transcription factors and *ghrelin* and *Ppy* or of beta-actin (*actb*) for the other hormones. Results are expressed as fold relative to levels in control pancreases (value of 1). Primer sequences are provided in S1 Table.

### Immunofluorescence and morphometric analysis

Pancreases were harvested, fixed 5h in 4% PFA and paraffin-embedded. Immunofluorescence staining was performed on 3 μm sections following standard procedures. Primary antibodies were: guinea pig anti-insulin (1:1000 dilution, Dako, Glostrup, Denmark), mouse anti-glucagon (1:500 dilution, Dako) and rabbit anti-somatostatin (1:500, Dako). Cye3 anti-guinea pig and Cy2 anti-rabbit or anti-mouse labeled secondary antibodies (1:500 dilution, Jackson Immunoresearch, Suffolk, UK) were used. Hoescht (1:500 dilution, Sigma-Aldrich) was employed as nuclear marker. Images were taken with a Leica DMR HC epifluorescence microscope and analyzed using Image J software. For morphometric measurements, 13 to 15 non-consecutive 3 μm thick sections (45 μm apart) were analyzed per pancreas.

### Intraperitoneal Glucose Tolerance Test (IPGTT)

In order to assess whole-body glucose tolerance, intraperitoneal glucose tolerance tests (IPGTT) were performed after 6 h food deprivation in Atoh8 Δ^panc^ and wild-type mice. The IPGTT was performed by administration of an injection of D-glucose (2 g/Kg body weight), and blood samples were collected from tail vein at 0, 15, 30, 60 and 120 minutes after injection. Glycaemia was measured at the same time points using a clinical glucometer and Accu-Check test strips (Roche Diagnostics, Switzerland). Plasma was obtained by blood centrifugation and kept at -80°C for insulin determination using a mouse insulin ELISA kit (Mercodia, Uppsala, Sweden).

### Islet isolation and *ex vivo* insulin secretion assay

Islets were isolated using collagenase digestion and Histopaque gradient (Sigma-Aldrich) purification as described elsewhere [16]. Separate batches of 8 freshly isolated islets were used to determine insulin secretion in static incubation assays as previously described [16]. Insulin was measured using a mouse insulin ELISA kit (Mercodia).

### Statistical analysis

Data are presented as mean ± standard error of the mean (SE). Statistical significance was tested using Student’s t-test.

## Results and Discussion

Atoh8 is expressed in multiple tissues during embryonic development [1–5]. In the developing pancreas, it is initially found in the mesenchyme and later in differentiating epithelial (endocrine and exocrine) cells [3]. Within the endocrine compartment, Atoh8 was suggested to modulate activation of the endocrine differentiation cascade, although this conclusion was based on gain-of-function experiments in cultured cells [3]. Given the ubiquitous expression of Atoh8, to address the autonomous role of Atoh8 in pancreatic cell differentiation *in vivo*, we sought to generate a pancreas-specific mouse knockout model for this gene. Hence, we designed a conditional allele to eliminate exon 1 that encodes 237 of 322 amino acids, including the Proline-rich region, the first helix and the basic domain of the bHLH domain of this transcription factor [17]. Despite the presence of *Atoh8* transcripts that comprise an occult exon in intron 1 spliced to exons 2 and 3, no open reading frame has been identified that includes the Atoh8 amino acids encoded by exons 2 and 3 [18]. It should be noted there have been described two germline *Atoh8* mouse models, which have yielded discrepant survival phenotypes. Thus, one model exhibited early embryonic lethality around gastrulation [3] whilst the other presented normal viability [18]. The reason for this inconsistency remains uncertain but it may be related to the different genetic strategies used to generate these lines, i.e. combined deletion of exons 1+2 (which encode the complete protein except for the stop codon which is coded by exon 3) [3] versus deletion of only exon 1 [18]. In view of these observations, our present strategy is expected to circumvent the potential effects of undefined regulatory sequences within intron 1 of the *Atoh8* locus.

**Fig 1:**
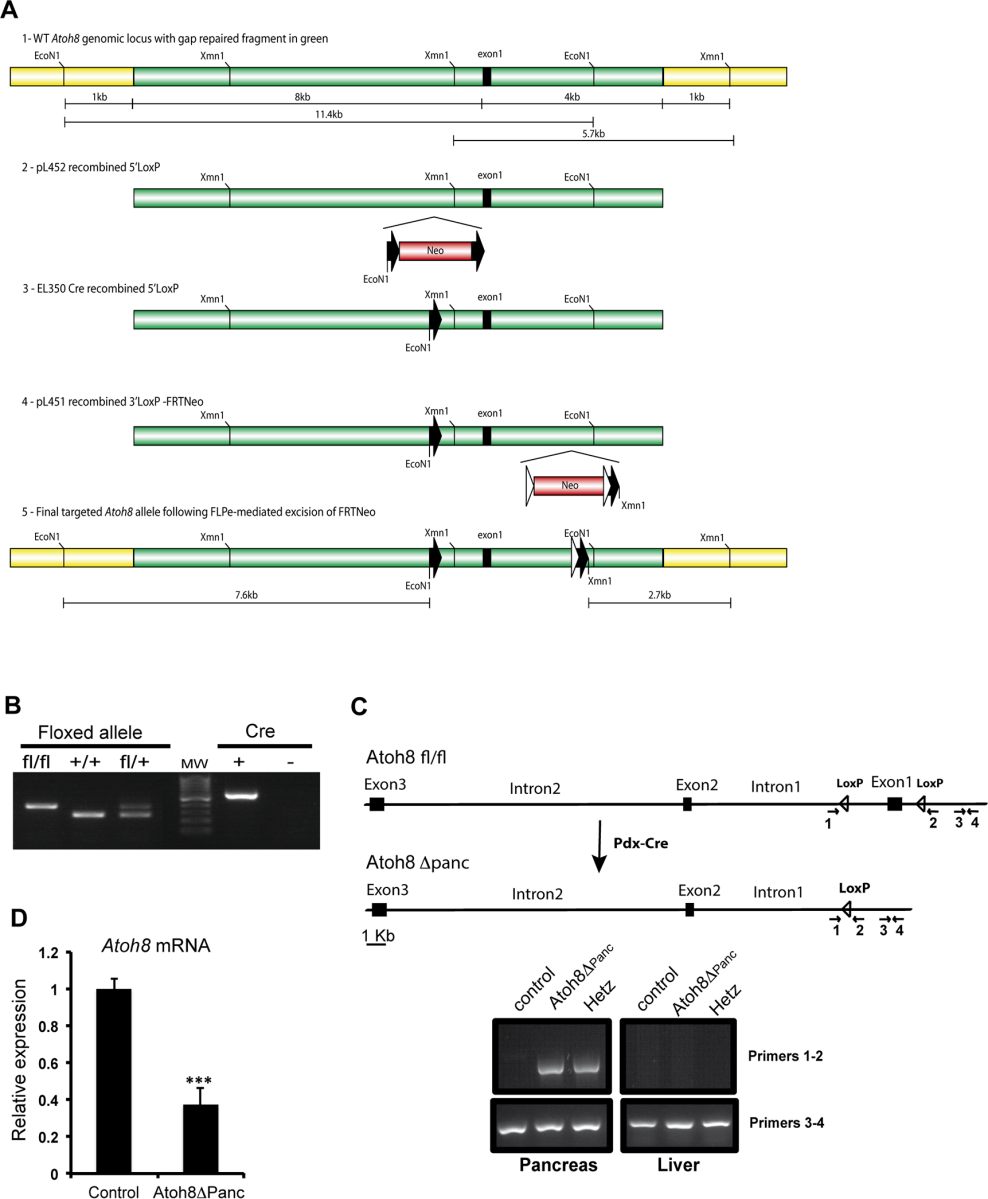
Generation of mice encoding a conditional allele of *Atoh8*. (A) Schematic representation of the generation of the *Atoh8* conditional allele construct. Detailed explanation is provided in Methods (B) Representative genotyping PCR amplification of *Atoh8* ^flox/flox^ wild-type and *Atoh8* ^flox/+^. PCR for detection of Cre recombinase is shown on the right; 100 pb molecular marker brightest line corresponds to 500 bp. Floxed allele 400 bp, wild type allele 290 bp; Cre 600 bp (C) Scheme and representative PCR amplification verifying Cre-mediated recombination of the *Atoh8* floxed allele in the pancreas and not in the liver from Atoh8 Δ^panc^ embryos at (E)15.5. Recombination mediated by Cre excises *Atoh8* exon1 that codifies for the 80% of the protein. The PCR amplification of the loxP region (primers 1 and 2) detects a band when recombination occurs; primers 3 and 4 are used as internal amplification control (D) *Atoh8* mRNA expression levels in (E)14.5 pancreata from Atoh8 Δ^panc^ and littermate controls as assessed by qRT-PCR. n = 7 per genotype; mean ± SE; ***p < 0.001 vs Control.

The targeting vector was constructed as described in Material and Methods and depicted in Fig 1A. Heterozygous (Flox/+) F1 mice were intercrossed to generate F2 offspring and the mice were genotyped by PCR of tail genomic DNA (Fig 1B). The floxed *Atoh8* mice were subjected to further characterization by mating them with the pancreas-wide deleter Pdx1-Cre line to generate Atoh8 Δ^panc^ mice. The Pdx1-Cre line drives Cre recombinase in the pancreatic epithelium starting at embryonic day (E)8.5 [14], which results in the elimination of the floxed gene in all pancreatic cell lineages, namely ductal, exocrine and endocrine. Tissue-specific recombination was verified by PCR genotyping of genomic DNA isolated from embryonic pancreas and liver (Fig 1C). Given that Atoh8 is first detected in the pancreatic epithelium after embryonic day (E)13.5, we confirmed that *Atoh8* mRNA levels were significantly reduced in the pancreas at (E)14.5 by qRT-PCR (Fig 1D). Remaining *Atoh8* mRNA expression (39%) detected in knockouts likely results from incomplete *Atoh8* elimination from the Pdx1+ expression domain (Pdx1-Cre is known to exhibit mosaic expression [19]) and from non-epithelial *Atoh8* expression [3]. In this regard, it is noteworthy that the relative proportion of mesenchyme to epithelium decreases as pancreas develops but it is still considerable at (E)14.5 [20]. Unfortunately, lack of working antibodies for immunostaining precluded confirmation of loss of Atoh8 protein in these mutants.

**Fig 2:**
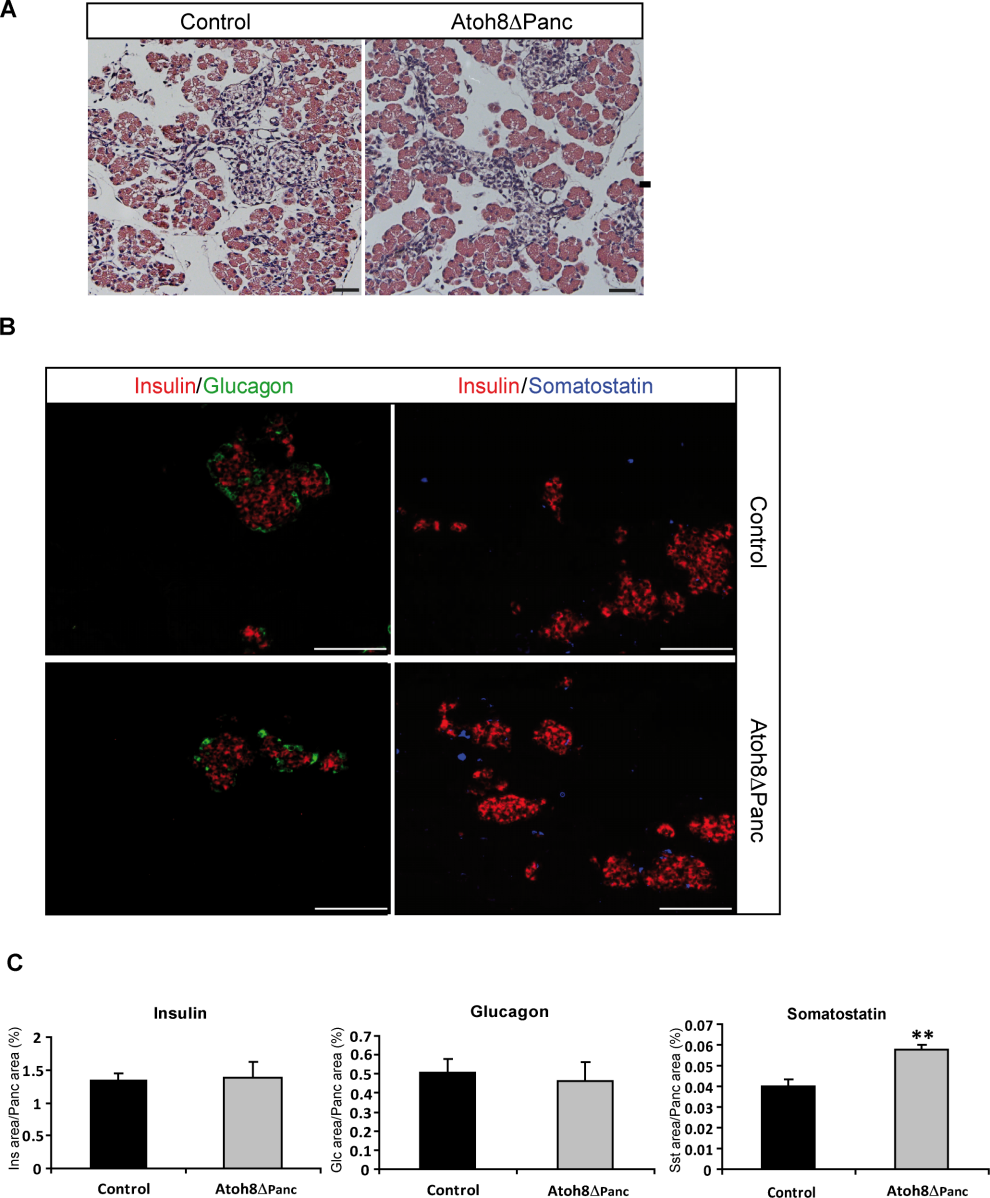
Pancreatic phenotype of Atoh8 Δ^panc^ mice at postnatal day 1. (A) Hematoxilin-eosin staining of pancreatic sections revealed similar organ structure in P1 Atoh8 Δ^panc^ and control littermates. Bars represent 50 **μ**M (B) Representative immunofluorescence staining for the major islets cell types (insulin in red, glucagon in green and somatostatin in blue) on pancreas tissue sections from P1 Atoh8 Δ^panc^ and control littermates. Bars represent 100 **μ**M (C) Morphometric quantification of hormone immunoreactive areas relative to total pancreatic area. n = 3; mean ± SE; **p < 0.01 vs Control.

Atoh8 Δ^panc^ mice were born at the expected mendelian ratio (analysis of F2 mice showed 24.5% of homozygous (flox/flox), 48% of heterozygous (flox/+) and 27.5% of wild-type (+/+)). Both flox/+ and flox/flox showed normal appearance and fertility. To assess the impact of *Atoh8* deficiency in pancreatic development, we examined Atoh8 Δ^panc^ mice at postnatal day 1 (P1). Knockout animals had similar body weight (control: 1.52±0.06 g, n = 10; Atoh8 Δ^panc^: 1.58±0.06 g, n = 8) and glycemia (control: 46.6±2.5 mg/dL; Atoh8 Δ^panc^: 50.8±3.8 mg/dL) to littermate controls. Pancreas weight (control: 7.7±0.6 mg; Atoh8 Δ^panc^: 9.85±0.09 mg; p = 0.07) and gross morphology as assessed by H&E staining were comparable between knockout and controls (Fig 2A), revealing that Atoh8 is not essential for pancreas formation or growth. We then analyzed the pancreatic endocrine compartment by immunofluorescence staining of the major islet hormones insulin (β-cells), glucagon (α-cells) and somatostatin (δ-cells). P1 knockout pancreata stained positive for all three hormones and islet architecture was normal as compared to controls (Fig 2B). Morphometric quantification of the endocrine cell area revealed increased δ-cell and comparable α- and β-cell areas between Atoh8 Δ^panc^ and controls (Fig 2C). An increase in δ-cell area was also observed using a different Pdx1-Cre transgenic line [21] (data not shown). Therefore, *Atoh8* deficiency affects δ-cells but has no apparent effect on α-cell and β-cell number.

**Fig 3:**
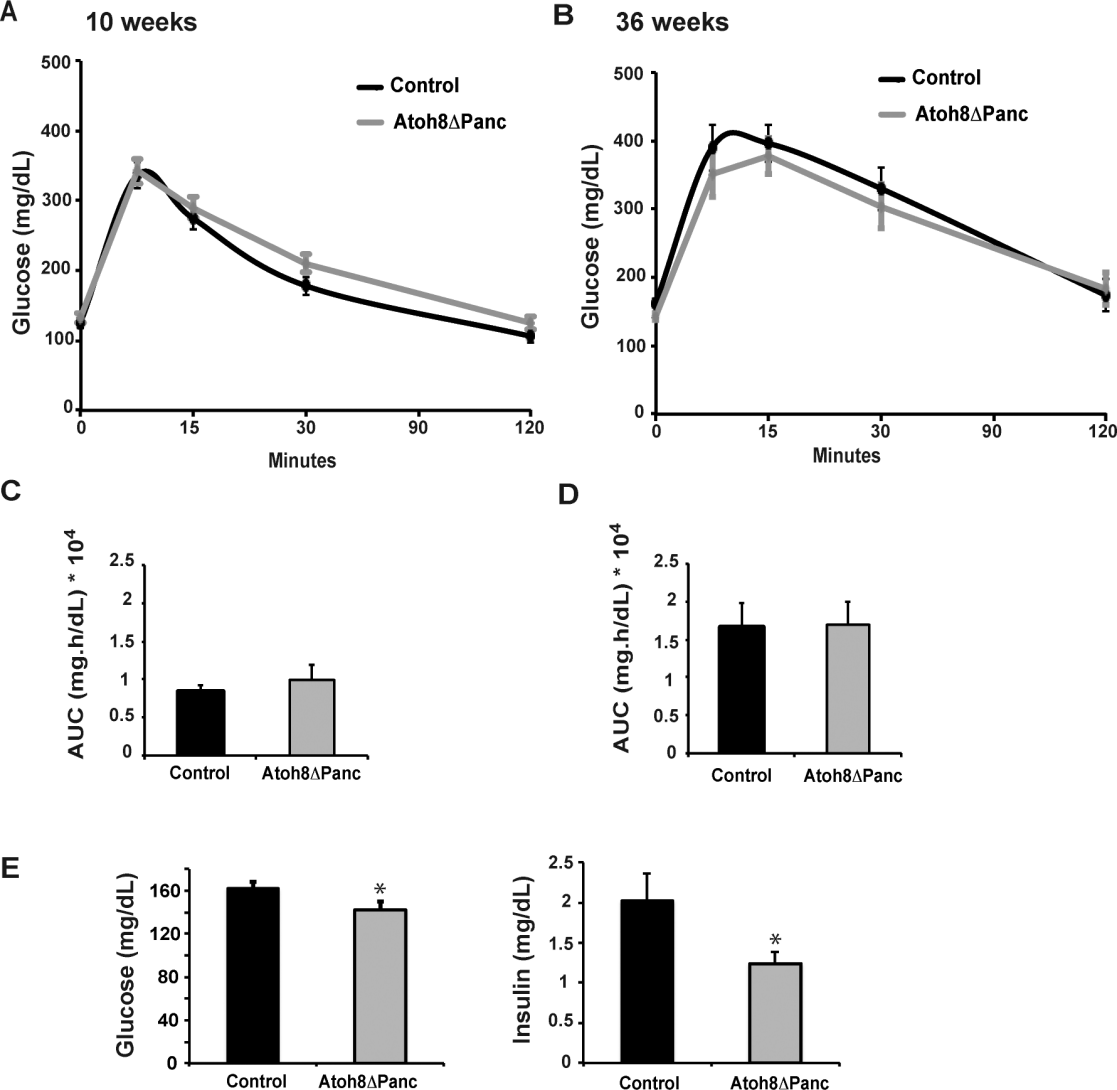
Glucose tolerance of adult Atoh8 Δ^panc^ mice. Intraperitoneal glucose tolerance tests were performed on 10-wk (A) and 36-wk-old (B) Atoh8 Δ^panc^ and control male mice fasted for 5 h. Area under the curve calculations for glucose tolerance tests in 10-wk-old (C) and 36-wk-old (D) mice. (E) Fasting glucose and insulin plasma levels of 36-wk-old control and Atoh8 Δ^panc^ mice. n = 12 (controls) and n = 14 (Atoh8 Δ^panc^); mean ± SE; *p < 0.05 vs Control.

**Fig 4:**
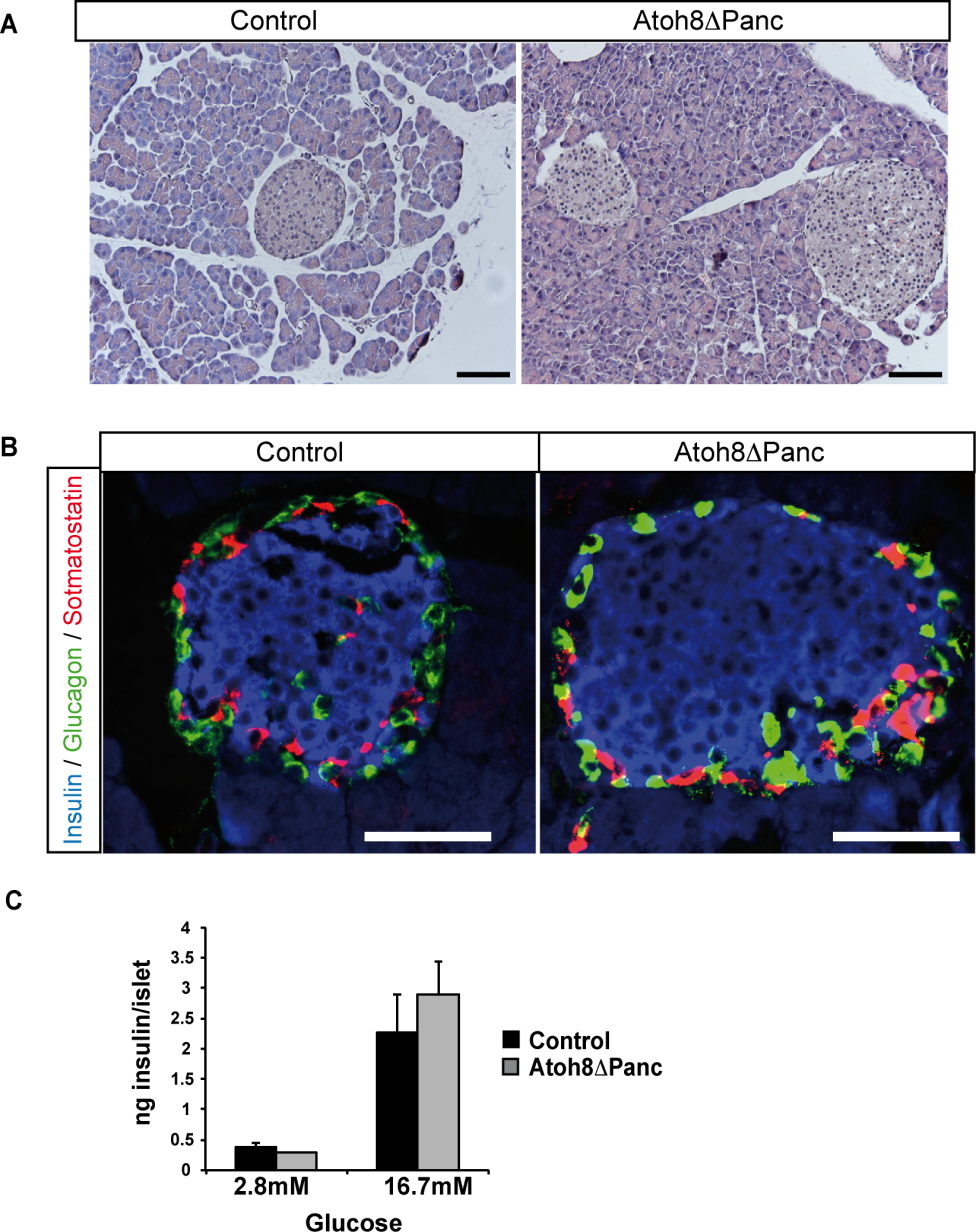
Pancreas morphology and islet insulin secretion in 36-week old Atoh8 Δ^panc^ mice. (A) Hematoxilin-eosin staining showed no apparent differences in pancreas gross morphology between 36-wk-old Atoh8 Δ^panc^ mice and controls. Scale bars 100 **μ**M (B) Immunofluorescence staining for insulin (blue), glucagon (green) and somatostatin (red) on pancreas sections from 36-wk-old Atoh8 Δ^panc^ mice and controls. Representative images demonstrate similar islet cell organization between mutants and controls. Scale bars 50 **μ**M (C) Glucose-induced insulin secretion in isolated islets from 36-wk-old Atoh8 Δ^panc^ mice and controls. n = 3 mice per genotype (3–4 independent islet batches per animal); mean ± SE.

Next, we studied whether this developmental phenotype had functional consequences for glucose homeostasis in adult animals and assessed glucose tolerance in 10-wk- and 36-wk-old Atoh8 Δ^panc^ male mice after an intraperitoneal glucose challenge. Body weight (10-wk control: 25.5±0.8 g; Atoh8 Δ^panc^: 26.1± 0.8 g and 36-wk control: 37.9±1 g; Atoh8 Δ^panc^: 35.4± 1 g) and glucose tolerance curves were indistinguishable between mutants and controls at both ages studied (Fig 3A–3D). Atoh8 Δ^panc^ females also displayed normal glucose tolerance (data not shown). Yet, we did observe a modest reduction in basal fasting glycemia and insulinemia in 36-wk-old male Atoh8 Δ^panc^ mice relative to controls (Fig 3E). We examined their pancreata and found that organ weight (control: 1.07±0.1 g *vs* Atoh8 Δ^panc^: 0.95±0.05 g) and gross morphology was similar to that of control animals of the same age (Fig 4A). Islet architecture was also comparable (Fig 4B). As endogenous islet somatostatin is thought to have a local effect on islet function acting as a paracrine tonic inhibitor of insulin and glucagon secretion, we assessed the ex *vivo* insulin secretory capacity of isolated islets using static incubation assays and found equivalent glucose-induced insulin secretion by Atoh8 Δ^panc^ and control islets (Fig 4C). Therefore, *Atoh8* deficiency in the fetal pancreas does not compromise whole body glucose tolerance or insulin secretion in adult islets, thus implying that the increment in δ-cell area observed in Atoh8 Δ^panc^ neonates appears to have no significant physiological impact in adult mice. In this regard, it is noteworthy that impaired δ-cell function in mice with pancreas-specific deletion of the homeodomain transcription factor Hhex does not compromise their glucose tolerance, although plasma insulin concentrations at basal or after a glucose challenge were found elevated [22]. These and our findings are consistent with the ascribed minor role of pancreatic somatostatin on the regulation of whole body glucose metabolism [23].

**Fig 5:**
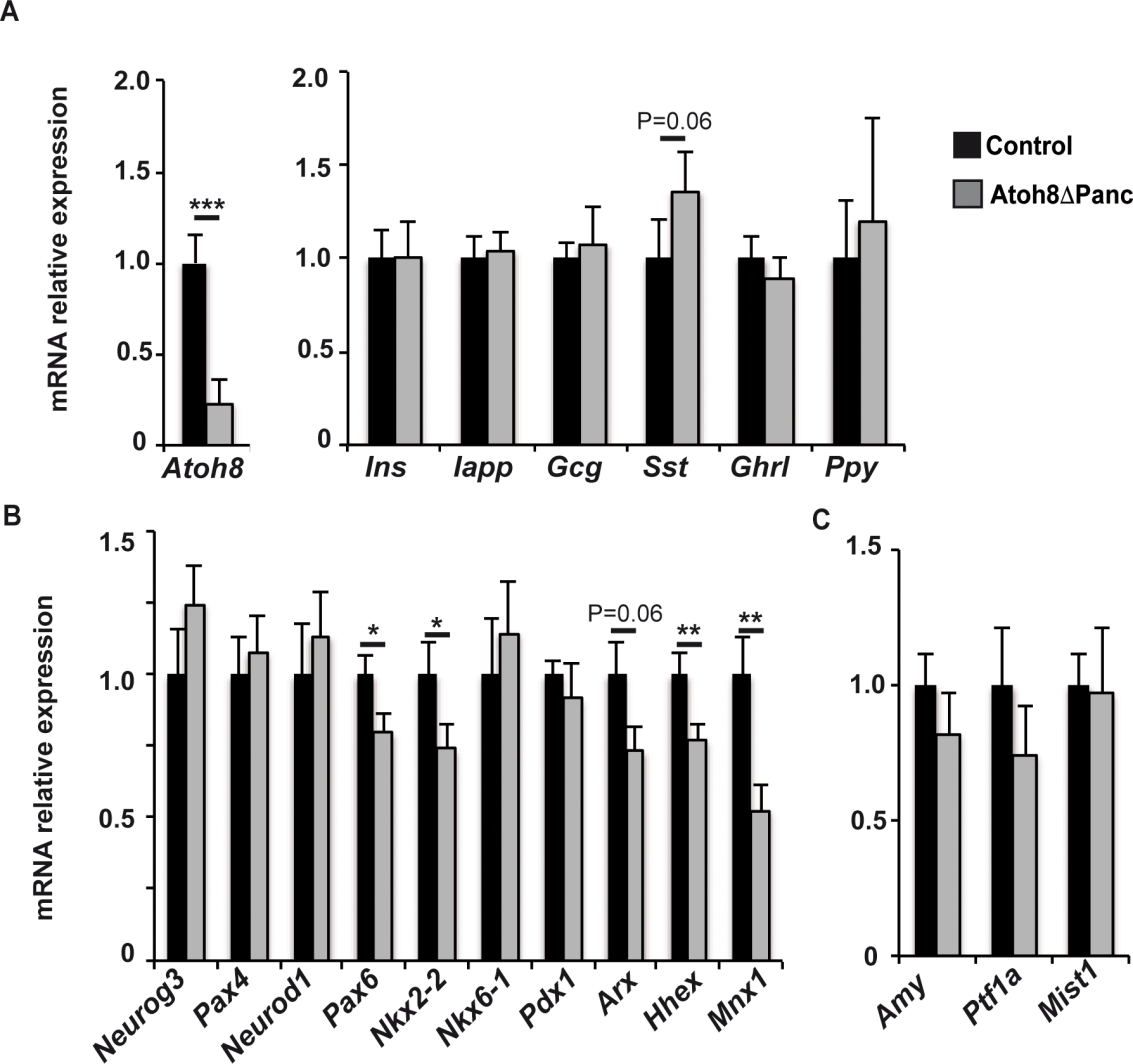
Gene expression levels of endocrine and exocrine differentiation markers in pancreases from Atoh8 Δ^panc^ mice at (E)15.5. Total pancreatic RNA was prepared from Atoh8 Δ^panc^ and control embryos at (E)15.5. Gene expression levels were assessed by qRT-PCR as described in Methods. Values are expressed relative to control pancreases, set at 1. Expression levels for *Atoh8* and endocrine cell markers (A), endocrine differentiation transcription factors (B) and exocrine genes (C). n = 10 per genotype from 4 independent litters; mean ± SE; * p< 0.05, ** p< 0.01;, *** p< 0.001 vs Control.

During development all pancreatic endocrine cell lineages derive from progenitor cells that transiently express the pro-endocrine transcription factor Neurogenin3 (Neurog3)[24]. Atoh8 is expressed in Neurog3+ cells at (E)15.5 but it is absent from late gestation endocrine cells [3], suggesting a transient role of this transcription factor during or immediately after Neurog3 expression. To gain understanding on how *Atoh8* loss impacted δ-cell formation, we assessed activation of the endocrine gene expression program by determining mRNA levels of a spectrum of early and late endocrine differentiation markers at (E)15.5. Despite a nearly 80% reduction of *Atoh8* transcript at this stage, endocrine gene expression remained largely unmodified. Thus, no significant differences were found in islet hormone expression, although the *somatostatin* message was slightly upregulated in mutants relative to controls (Fig 5A). On the other hand, mRNA levels for the endocrine differentiation transcription factors *Neurog3*, *Pax4*, *NeuroD1* and *Nkx6*.*1* were comparable, whereas transcripts for *Pax6*, *Nkx2*.*2*, *Arx*, *Hhex* and *Mnx1* were reduced in Atoh8 Δ^panc^ relative to controls (Fig 5B). To date, Hhex is the only genetic factor shown to specify δ-cell fate during pancreatic development [22]. Yet, *hhex* transcripts were decreased by 20% in mutants, indicating that enhanced δ-cell formation did not correlate with upregulation of this transcription factor, at least at this developmental stage. Remarkably, it has been recently reported that inactivation of *Mnx1* in endocrine progenitors leads to increased δ-cell allocation to the detriment of β-cell differentiation [25]. Hence, our finding of reduced *Mxn1* transcript levels in Atoh8 Δ^panc^ embryos would be compatible with increased δ-cell differentiation in these mutants. Furthermore, it is interesting to note that simultaneous deletion of both Nkx2.2 and Arx in mice leads to the deregulation of *somatostatin* expression [26, 27]. Thus, it is plausible that combined changes in levels of multiple transcriptional regulators results in increased δ-cell numbers in *Atoh8* mutants. Nonetheless, these changes appear to be insufficient to affect the formation of the more abundant islet cell types α and β. These data argue that Atoh8 contributes mildly to activation of endocrine differentiation *in vivo* and implies that this transcription factor likely acts as a modulator rather than a promoter of differentiation in the pancreas. Although we cannot rule out compensatory effects by other genes, our current *in vivo* findings are consistent with prior results in a cell-based endocrine differentiation model demonstrating lack of pro-endocrine activity of Atoh8 [17].

Further investigations on Atoh8 at a molecular level may help shed light on the function of this transcription factor during activation of the pancreatic endocrine program. In any case, a better comprehension of δ-cell differentiation is required to discern the molecular pathways modified by *Atoh8* ablation that are being affected in Atoh8 Δ^panc^ mutants. Further, given that (i) Atoh8 is first detected in the pancreatic epithelium after (E)13.5 [3], (ii) Neurog3+ endocrine progenitors acquire the capacity to differentiate to δ-cells from (E)14.5 [28] and (iii) δ-cells are first immunodetected at (E)15.5 [29], one possibility is that the specific impact on δ-cell formation is related to a timing effect. The availability of this floxed model warrants further studies using temporally regulated Cre transgenic lines to address this as well as other questions. Finally, as Atoh8 is also expressed in the differentiating exocrine compartment [29], we determined expression levels for amylase and the acinar-specific transcription factors Ptf1a and Mist1. In agreement with normal pancreatic morphology and size, these genes were similarly expressed in Atoh8 Δ^panc^ and their littermate controls at (E)15.5 (Fig 5C).

The floxed *Atoh8* mice will provide a valuable tool to elucidate time or cell-specific functions of Atoh8 during development and in the adult. Given the multiple cellular contexts where Atoh8 has been reported to participate, availability of this model guarantees future studies aimed at investigating the role of this little-known transcription factor in multiple organs and tissues *in vivo*.

## Supporting Information

S1 Table List of oligonucleotides used for genotyping and qRT-PCR. (PDF)
