# Supplementary material for: Generation of a Conditional Allele of the Transcription Factor Atonal Homolog 8 (Atoh8)
Source: PLoS One. 2016 Jan 11;11(1):e0146273. doi: 10.1371/journal.pone.0146273 (PMC4708992; doi:10.1371/journal.pone.0146273)
Supplement: S1 Table — (PDF) [file pone.0146273.s001.pdf]

Table S1. List of oligonucleotides used for genotyping and qRT-PCR

Gene	Sequence	Aplication
**Pdx1-Cre**	5’ CCCCAGAAATGCCAGATTACG<br>3’ CGTAGAGAGTCCGCGAGCCA	**Genotyping**
**Math6-LoxP**	5’ ATTGGAGGAAGGCTCGGTGAA<br>3’ TTGGCATTCGTCGTGCTGTC	**Genotyping**
**Math6 (Primer1 and primer2)**	5’AGCGTCCTTTGGGTAGATATATTCAACAC<br>3’ATTGGAGGAAGGCTCGGTGAA	**Pancreas recombination**
**Math6 (control: Primer3 and primer4)**	5’AAGAACTGAACGGCCTCAAGAAGC<br>3’GATCGGGAACGGTTGTGTCC	**Pancreas recombination**
**Cre**	5’ CTGCAGCACCTCTTCCAGGT<br>3’ TCGGATCCGCCGCATAACCA	**qPCR**
**Math6**	5’ AGCCAAGAAACGGAAGGAGTG<br>3’ TCTGGCAGCATCTTGAGGAAG	**qPCR (exon 1)**
**Somatostatin**	5’ACCCCAGACTCCGTCAGTTTC<br>3’ATCATTCTCTGTCTGGTTGGGC	**qPCR**
**Insulin (1+2)**	5’ AGCGTGGCTTCTTCTACACACC<br>3’ CCAGCTCCAGTTGTGCCACT	**qPCR**
**Glucagon**	5’ AGGAATTCATTGCGTGGCTG<br>3’ CAATGGCGACTTCTTCTGGG	**qPCR**
**Ghrelin**	5’ CCATCTGCAGTTTGCTGCTA<br>3’ GCTTGTCCTCTGTCCTCTGG	**qPCR**
**Neurog3**	5’TTCTCATCGGTACCCTTGCTG<br>3’GCAGACTCACCAGGAAGTATGG	**qPCR**
**Pdx1**	5’ CCCCAGTTTACAAGCTCGCT<br>3’ CTCGGTTCCATTCGGGAAAGG	**qPCR**
**Arx**	5’ TTCCAGAAGACGCACTACCC<br>3’ TCTGTCAGGTCCAGCCTCAT	**qPCR**
**NeuroD1**	5’ GGATCAATCTTCTCTTCCGGTG<br>3’ TGCGAATGGCTATCGAAAGAC	**qPCR**
**Nkx2.2**	5’ GAACAGCAAGCTAGCCGAGG<br>3’ CGATCAGTCCATATAAGGCTGG	**qPCR**
**Nkx6.1**	5’ TGGACAGCAAATCTTCGCCCTG<br>3’ TGTTGTAATCGTCGTCATCCTC	**qPCR**
**Pax4**	5’ GAGTACCCTGCTCTTTTTGCC<br>3’ ACTCGATTGATAGAGGACACACT	**qPCR**
**Pax6**	5’ TACCAGTGTCTACCAGCCAAT<br>3’ TGCACGAGTATGAGGAGGTCT	**qPCR**
**Hhex**	5’ AGCTCAGTGAGAGACAGGTCA<br>3’ GTGTCCAAACTGTCCAACGC	**qPCR**
**Ppy**	5’ ACCCAGGCGACTATGCGACACC<br>3’AGCTCCCTGCCTTCAGCTCCAG	**qPCR**
**Actb**	5’ AAATCGCTCCTGTCGAGTCGCG<br>3’ ACGACCAGCGCAGCGATATCG	**qPCR**
**Tbp**	5’ ACCCTTCACCAATGACTCCTATG<br>3’ ATGATGACTGCAGCAAATCGC	**qPCR**
**Amylase**	5’ TGGCGTCAAATCAGGAACATG<br>3’ AAAGTGGCTGACAAAGCCCAG	**qPCR**
**Ptf1a**	5’ ACAAGCCGCTAATGTGCGAGA<br>3’ TTGGAGAGGCGCTTTTCGT	**qPCR**
**Mist1**	5’ GCTGACCGCCACCATACTTAC<br>3’ TGTGTAGAGTAGCGTTTGCAGG	**qPCR**
**Mnx1**	5’ GTCTACTGCGGGCATGATCC<br>3’ CACCTCAAAACGCTTGGGTC	**qPCR**
